# The ChpR transcriptional regulator of *Sinorhizobium meliloti* senses 3,5,6-trichloropyridinol, a degradation product of the organophosphate pesticide chlorpyrifos

**DOI:** 10.1099/acmi.0.000297

**Published:** 2021-12-15

**Authors:** Nathan D. McDonald, Courtney E. Love, Henry S. Gibbons

**Affiliations:** ^1^​ United States Army Combat Capabilities Development Command – Chemical Biological Center, Aberdeen Proving Ground, Maryland, USA

**Keywords:** chlorpyrifos, biosensor, 3,5,6-trichloropyridinol, organophosphate pesticide, degradation products

## Abstract

The global use of organophosphate insecticides (OPPs) and the growing concern of off-target side effects due to OPP exposure has prompted the need for sensitive and economical detection methods. Here we set out to engineer a previously identified OPP responsive transcription factor, ChpR, from *Sinorhizobium melilotii* to respond to alternative OPPs and generate a repertoire of whole-cell biosensors for OPPs. The ChpR transcription factor and cognate promoter P*_chpA_,* have been shown to activate transcription in the presence of the OPP chlorpyrifos (CPF). Utilizing a GFP reporter regulated by ChpR in a whole-cell biosensor we found that the system responds significantly better to 3,5,6-trichloro-2-pyridinol (TCP), the main degradation product of CPF, compared to CPF itself. This biosensor was able to respond to TCP at 390 nM within 4 h compared to 50 µM of CPF in 7 h. The ChpR-P*_chpA_*, and the activating ligand TCP, were able to regulate expression of a kanamycin resistance/sucrose sensitivity (*kan/sacB*) selection/counterselection module suitable for high throughput mutagenesis screening studies. The ability to control both GFP and the *kan/sacB* module demonstrates the utility of this reporter for the detection of CPF affected areas. The ChpR-P*_chpA_* system serves as an additional positive regulator switch to add to the growing repertoire of controllers available within synthetic biology.

## Introduction

The potential of pollutants entering the environment through a variety of mechanisms can have a wide array of adverse side effects [1]. One major source of pollution is a result of agricultural processes including the application of pesticides and fertilizers to crops [2, 3]. One group of insecticides in particular, the organophosphate pesticides (OPPs), exhibit toxic effects on humans and a variety of animals [2, 4–10]. A rise in awareness surrounding pollutants and OPPs has prompted the development of economical and field deployable methodologies to replace the expensive and labour intensive analytical methods currently needed to detect OPPs [11, 12]. Whole-cell and cell-free biosensors have proven to be simple, inexpensive, portable and sensitive alternatives for substrate detection [12–15]. Many of these biosensors are derived from prokaryotes and rely on transcription factor-based regulatory circuits that have been synthetically engineered to produce a signal in response to a chemical inducer. Examples of transcription-factor-based biosensors for the detection of environmental pollutants include aromatic compound detection modules, sensors for the detection of heavy metals as well as antibiotic detection (reviewed in [15]).

**Fig. 1.**
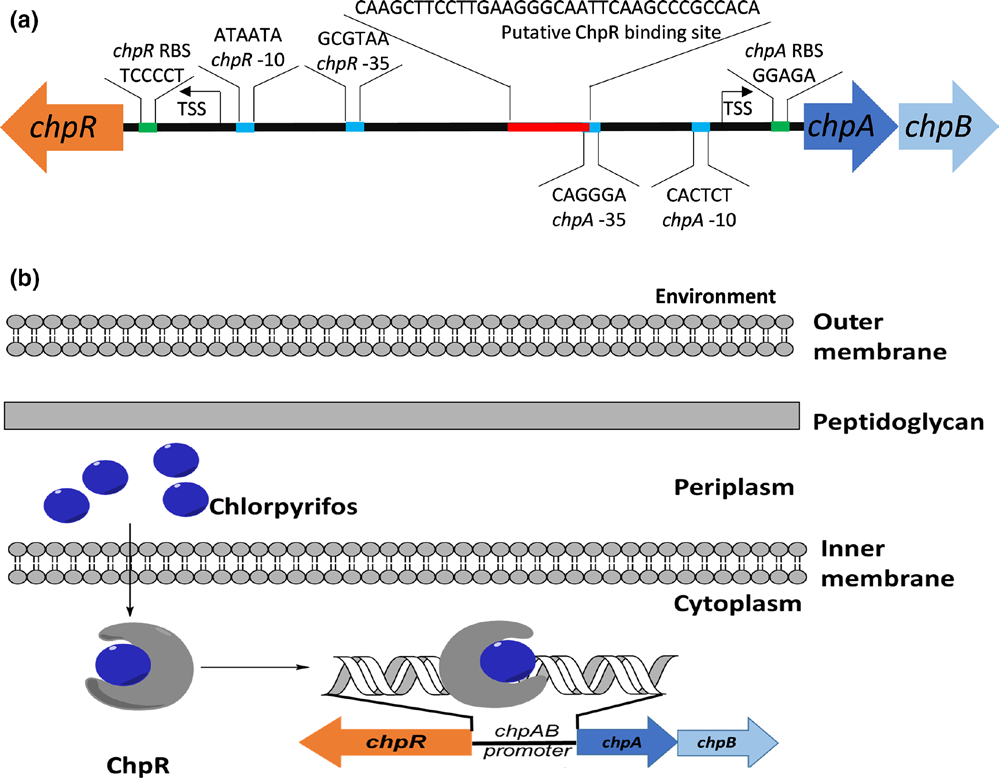
(a) Arrangement of *chpR* and p*chpA* in *Sinorhizobium meliloti *. Previous work has demonstrated ChpR binds to P*_chpA_* to activate transcription in the presence of chlorpyrifos. (b) Predicted model of chlorpyrifos binding ChpR in the bacterial cytoplasm to activate transcription through direct binding to P*_chpA_*.

Previous work by Whangsuk and co-workers identified a transcription factor, ChpR, belonging to the CadC family of regulators from *Sinorhizobium melilotii* that activated transcription in response to the OPP chlorpyrifos (CPF) [16]. It was hypothesized that CPF binds directly to ChpR resulting in activation of the cognate promoter P*_chpA_* [16]. It was found that ChpR binds directly to a promoter, P*_chpA_*, and activates transcription in the presence of CPF and that ChpR was required for transcription indicating it is a positive regulator (Fig. 1) [16]. The researchers utilized ChpR and its responsive element to create a whole-cell biosensor in *Escherichia coli* that could specifically detect CPF on a linear range from 25 to 500 nM [17]. This system has since been improved through random mutagenesis and the engineered ChpR reporter was integrated into the *E. coli* genome [18]. This iteration of the ChpR biosensor was able to detect CPF at 5 nM [18].

## Methods

### Bacteria and culture conditions

The *E. coli* strains were routinely grown aerobically with aeration (225 r.p.m.) at 37 °C in Luria–Bertani (LB) broth (Fisher Scientific, Waltham, MA). Stock solutions of CPF (Sigma Aldrich) and TCP (Sigma Aldrich) were made at 200 mM in DMSO (Sigma Aldrich) and used for serial dilutions in DMSO to obtain desired concentration. For evaluating activation of pChprD reporter, strains were grown overnight in LB with aeration at 37 °C, supplemented with the indicated final concentrations of either chlorpyrifos, TCP, or DMSO. After overnight growth, 1 ml of culture was centrifuged at 5000 ***g*** for 5 min to pellet the bacteria. The pellets were washed twice in PBS and resuspended in 1 ml of PBS. A total of 200 µl of each culture condition was added in triplicate to a 96-well plate (Corning) and optical density (Ab. 600 nm) and GFP fluorescence (Ex. 485 Em. 523) were measured on a BioTek plate fluorescent plate reader. Antibiotics were used at the following concentrations: ampicillin (100 ug ml^−1^) or Kanamycin (50 µg ml^−1^).

### Reporter plasmid construction

The sequences for the coding regions for *chpR* and the upstream sequence from *chpA* containing the promoter (P*_chpA_*) from *Sinorhizobium melilotii* were obtained from NCBI genome database along with the sequences for the kanamycin/sacB cassette. ChpA in this construct is replaced beginning with its ATG start codon with the kanamycin-resistance element. The three bases prior to the start are replaced with CAT to form an *Nde*I site to facilitate subsequent manipulation. The pChprKS reporter was constructed and purchased from ATUM (Newark, CA) and propagated in *E. coli* DH5α. To construct the fluorescent reporter pChpRD, the Kan/Sac cassette was excised via restriction digest with enzymes NdeI and SacI for 2 h at 37 °C and replaced with Dasher GFP via Gibson Assembly (NEB) and transformed into *E. coli* DH5α. Successful clones were identified via restriction digest confirmation.

### Fluorescent time-course analyses

Overnight cultures of *E. coli* +pChpRD were grown in LB at 37 °C with shaking. The cultures were diluted 1 : 50 in 200 µl of LB supplemented with ampicillin or either CPF, TCP or DMSO at the desired concentration. The plate was incubated 37 °C with shaking in a Biotek fluorescent plate reader for 8 h. The growth and fluorescence of the system were monitored by measuring OD_600_ and GFP fluorescence every 10 min.

### Characterization of Kan/sacB selectable reporter

The *E. coli* +pChpRKS reporter was grown overnight in LB supplemented with 100 uM of CPF or TCP or the equal volume of DMSO carrier. To evaluate induced kanamycin resistance, the overnight cultures were serially diluted in PBS and plated on LB agar plates containing kanamycin (50 µg ml^−1^) either with or without 100 µM of the cognate inducer molecule. Following the plating, the plates were incubated overnight at 37 °C and photographed. Sucrose sensitivity was determined in a similar fashion with the induced or control overnight cultures of *E. coli* +pChpRKS being serially diluted and plated on LB agar plates containing 8 % w/v sucrose and either 100 µM of inducer or DMSO carrier control. The plates were incubated overnight at 37 °C prior to being photographed.

## Results

**Fig. 2.**
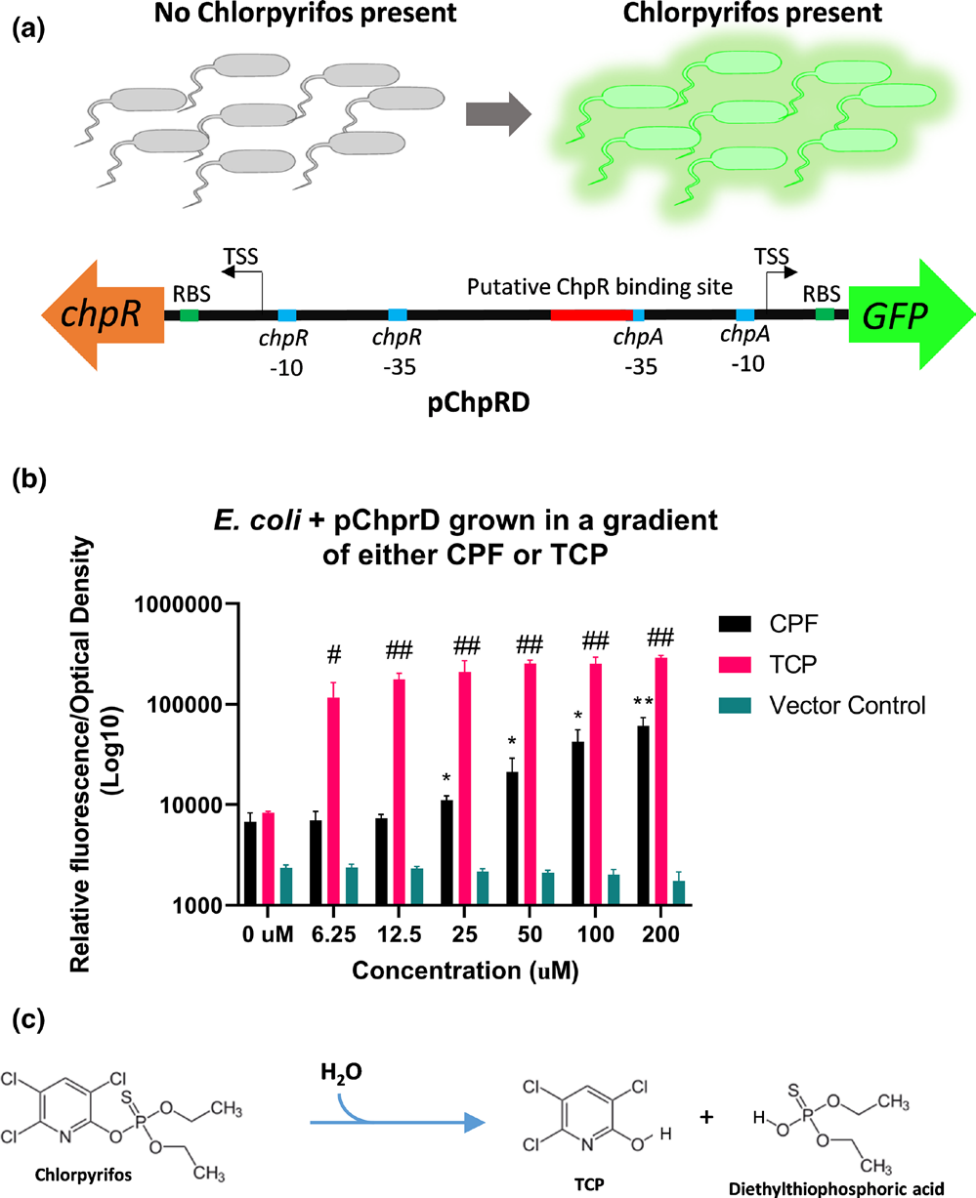
(a) The coding region of *chpR* and P*_chpA_* were cloned to control GFP and transformed into *E. coli* DH10B to create a whole-cell chlorpyrifos biosensor designated pChpRD. (b) pChpRD and a vector control was grown in LB supplemented with a gradient of either CPF or TCP and the specific fluorescence (RFU/OD) was measured after 24 h. (c) Hydrolysis of CPF to TCP and diethylthiophosphoric acid in the presence of water. *P<0.05 **P<0.01. #*P*<0.05 ##*P*<0.01. Data shown represent at least three biological replicates and three technical replicates.

With ChpR able to detect CPF in the nanomolar range with a high degree of specificity, we set out to utilize synthetic biology and a directed evolution campaign to reengineer ChpR to respond to alternative OPPs resulting in a library of whole-cell biosensors for this dangerous class of chemicals. First, the coding region of *chpR* and the cognate promoter region P*_chpA_* were cloned to control the expression of GFP (*Dasher* variant, ATUM), and the entire construct (designated pChpRD) was transformed into *E. coli* to generate a whole-cell biosensor designated as *E. coli* +pChpRD (Fig. 2a). To validate the functionality of this biosensor, the strain was grown overnight in LB supplemented with a dose gradient of CPF ranging from 0 to 200 µM and specific fluorescence (RFU/optical density) was determined in a 96-well plate on a BioTek plate reader. Across the concentrations 25–200 µM of CPF, the pChpRD biosensor was significantly activated compared to the no treatment condition confirming that this system does respond to CPF (Fig. 2b). The initial reports of ChpR found that this system responded specifically to CPF and was not activated by other OPPs [19]. With the goal of engineering ChpR to respond to alternative OPPs, we grew the pChpRD-transformed strain in the presence of parathion, methyl-parathion, diazinon, and malathion and as expected did not observe any GFP activation over the control (data not shown).

Chlorpyrifos is a highly non-polar compound and in the environment it readily hydrolyzes to its main degradation product 3,5,6-trichloro-2-pyridinol (TCP) (Fig. 2c) [20, 21]. We evaluated the ability of TCP to activate the pChpRD biosensor and found it to be a potent inducer of the system from 6.25 to 200 µM compared to the no treatment condition (Fig. 2b). Following the overnight growth in LB supplemented with a concentration gradient of TCP, the specific fluorescence was significantly greater with TCP induction compared to CPF inductions (Fig. 2b). It is important to note that while ChpR was identified from *Sinorhizobium meliloti* as a CFP responsive element, the native ligand for ChpR in the environment has not been confirmed. Our observation that TCP is a more potent activator of ChpR may suggest that CPF or TCP are not the native ligand and that ChpR is likely not suitable for engineering to respond directly to alternative OPPs. We speculate that the activation of ChpR we observe by CPF is a result of hydrolysis in the growth medium to TCP.

**Fig. 3.**
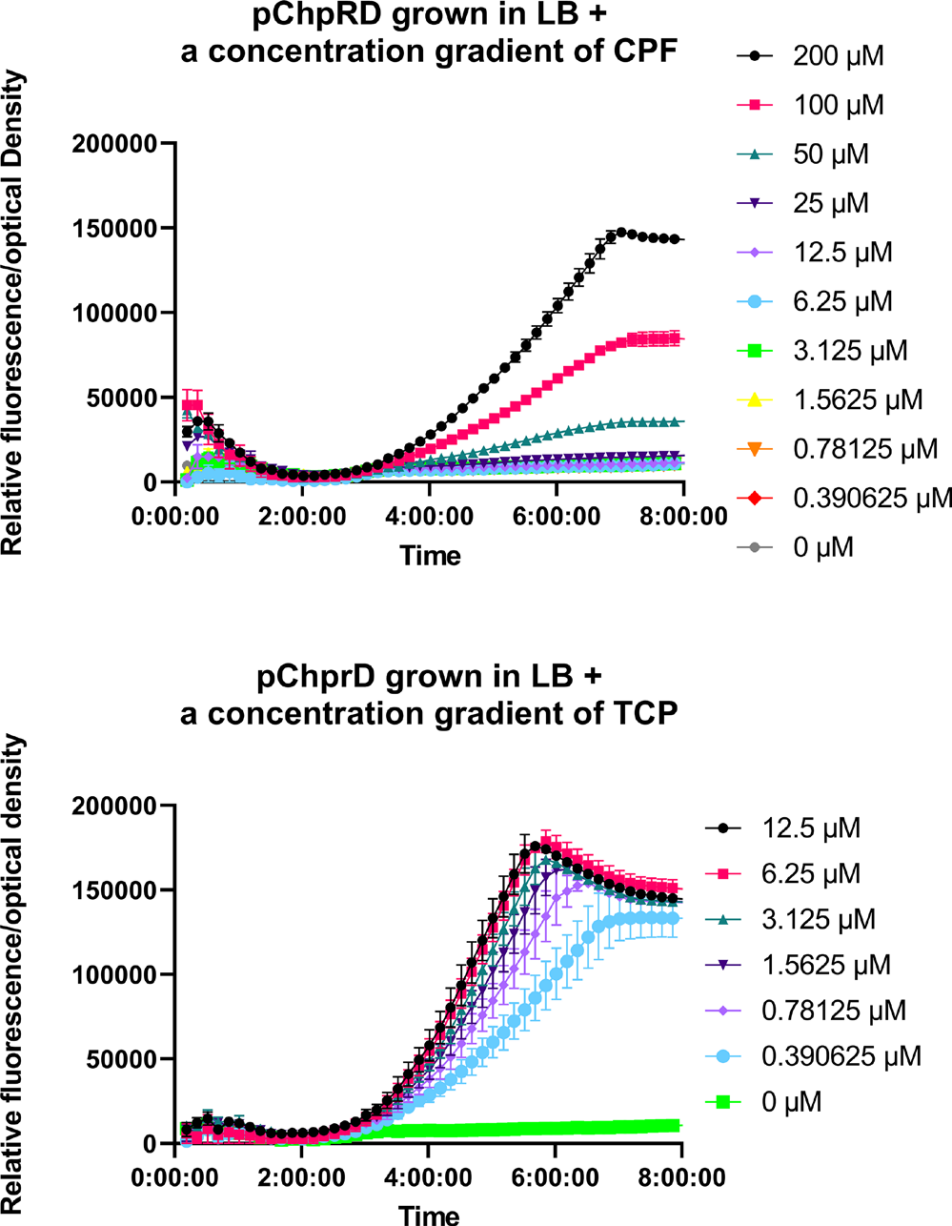
(a) Specific fluorescence growth response of pChpRD grown in LB with a concentration gradient of CPF. (b) Specific fluorescence of pChpRD grown in LB with increasing concentrations of TCP.

In order to better assess the activation of ChpR and responsiveness of the system, we measured the response rate through a growth curve analysis. The pChpRD biosensor was grown overnight in LB in the absence of any inducer. The cultures were subcultured 1 : 50 in 96-well plates in LB containing a concentration gradient of either CPF or TCP and the optical density and fluorescence were monitored every 10 min (Fig. 3). Across 8 h, the pChpRD biosensor was activated by 50–200 µM of CPF, with activation initiating at ~3 h (Fig. 3a). Conversely, when grown in the presence of TCP, the sensor was activated within the same 8 h at a TCP concentration of 0.39 µM, again with activation of the system initiating at ~3 h (Fig. 3b). These data further demonstrating that ChpR responds more robustly to TCP.

**Fig. 4.**
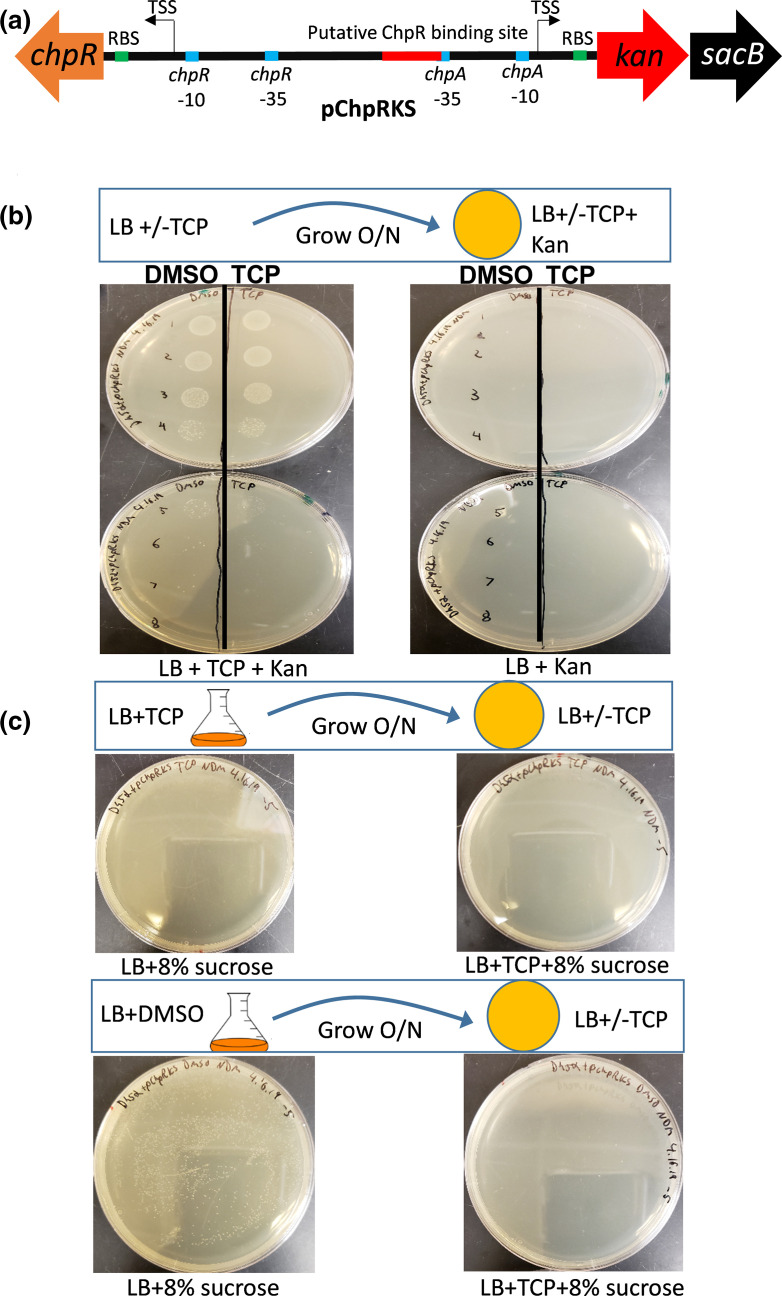
(a) The chpR and P*_chpA_* regions were constructed to control a *kan/sacB* cassette designated pChpRKS. (b) pChpRKS was grown overnight in LB +/-TCP and serially diluted and plated on LB agar supplemented with Kan+/-TCP. Vertical line separates the overnight growth conditions numbers indicate dilution factor. (c) pChpRKS was grown overnight +/-TCP and plated on LB +/-TCP and+/-8 % sucrose.

Having established TCP as a potent inducer of ChpR we set out to develop a selection module for directed evolution and protein engineering investigations. Utilizing the same design as described above for the GFP reporter, we created a selection/counterselection module under the control of ChpR-P*_chpA_*. Based on the work of Garmendia and co-workers [22], the module consists of a bicistronic kanamycin (*kan*) resistance cassette paired with the *sacB* sucrose sensitivity cassette. The resulting module was named pChpRKS (Fig. 4a). With this module, when the system is activated, the bacterium becomes resistant to kanamycin and sensitive to sucrose. When the system is inactive, the bacterium is kanamycin sensitive and sucrose resistant. Together, this module allows for a high throughput screen of activator mutants by identifying those that are strongly induced in the presence of the desired induction ligand while selecting against mutants that are constitutively expressed.

We tested the functionality of pChpRKS by growing the system overnight in LB supplemented with either 100 µM TCP or DMSO, the solvent we used to deliver TCP and the other OPPs. The cultures were serially diluted and spot plated on LB agar supplemented 50 µg ml^−1^ kanamycin and either 100 µM TCP or DMSO control. Kanamycin resistance was observed on the plates containing TCP regardless of whether the pChpRKS was grown overnight in TCP or DMSO, suggesting that the TCP in the plates was sufficient to induce Kan resistance (Fig. 4b). Conversely when no TCP was present in the plates, no Kan resistance was observed regardless of whether the reporter module was grown overnight in the presence of TCP. Ultimately this result demonstrates that TCP is sufficient to activate the ChpR and consequently induces Kan resistance (Fig. 4b).

Next we tested the same module for the ability to activate sucrose sensitivity through the induction of the *sacB*. The pChpRKS was again grown overnight in LB with or without TCP as an inducer. The cultures were plated on LB agar plates supplemented with 8 % w/v sucrose and either TCP or DMSO. In this case, growth on sucrose plates was only observed when TCP was absent from initial overnight growth or the plates (Fig. 4c). These data demonstrate that the *sacB* gene of the selection/counterselection module is inducible through TCP and ChpR. The pChpRKS reporter module was also tested with 100 µM CPF as the inducer and the system was not activated (data not shown). This *kan/sacB* cassette can be paired with other controllers as a tool for high throughput screening of enhanced activation of the system or for identifying mutants that respond to ligands of interest.

## Conclusions

This study demonstrated that the transcription factor ChpR is activated by the CPF degradation product TCP. The ability to identify TCP in a whole-cell biosensor represents a sensitive and economical method for screening contaminated agriculture and water run-off systems for evidence of contamination with TCP, and, indirectly, with CPF . It is not clear if the previous reports that ChpR senses CPF in actuality resulted from CPF hydrolysis and consequent secondary detection of TCP [17–19]. It is also important to note the limitations of using a biosensor, in which the native ligand has not been definitively identified, as it opens the door for false positives. Finally, we note the TCP is also a degradation product of the systemic herbicide triclopyr [23], which would limit its use to detect TCP as a surrogate for CPF. Future studies on ChpR should be focused on determining the natural ligand and specifically the active site interactions with TCP/CPF.

Furthermore we have demonstrated that ChpR-p*_chpA_* is a robust positive regulator in the presence of TCP with a high dynamic range. This system adds to the growing repertoire of genetic control modules available to the field of synthetic biology. We have also demonstrated a selection/counterselection module that has the potential for use in the screening of gain-of-function mutations or screening libraries for a desired response. This module is highly sensitive with no observable background observed under the conditions tested.
